# Functional and structural dissection of the tape measure protein of lactococcal phage TP901-1

**DOI:** 10.1038/srep36667

**Published:** 2016-11-08

**Authors:** Jennifer Mahony, Mona Alqarni, Stephen Stockdale, Silvia Spinelli, Marine Feyereisen, Christian Cambillau, Douwe van Sinderen

**Affiliations:** 1 School of Microbiology, University College Cork, Cork, Ireland; 2 APC Microbiome Institute, University College Cork, Cork, Ireland; 3 Architecture et Fonction des Macromolécules Biologiques, Aix-Marseille Université, Campus de Luminy, Marseille, France; 4 Architecture et Fonction des Macromolécules Biologiques, Centre National de la Recherche Scientifique (CNRS), Campus de Luminy, Marseille, France

## Abstract

The tail tape measure protein (TMP) of tailed bacteriophages (also called phages) dictates the tail length and facilitates DNA transit to the cell cytoplasm during infection. Here, a thorough mutational analysis of the TMP from lactococcal phage TP901-1 (TMP_TP901-1_) was undertaken. We generated 56 mutants aimed at defining TMP_TP901-1_ domains that are essential for tail assembly and successful infection. Through analysis of the derived mutants, we determined that TP901-1 infectivity requires the N-terminal 154 aa residues, the C-terminal 60 residues and the first predicted hydrophobic region of TMP_TP901-1_ as a minimum. Furthermore, the role of TMP_TP901-1_ in tail length determination was visualized by electron microscopic imaging of TMP-deletion mutants. The inverse linear correlation between the extent of TMP_TP901-1_-encoding gene deletions and tail length of the corresponding virion provides an estimate of TMP_TP901-1_ regions interacting with the connector or involved in initiator complex formation. This study represents the most thorough characterisation of a TMP from a Gram-positive host-infecting phage and provides essential advances to understanding its role in virion assembly, morphology and infection.

Bacteriophages are the most abundant and genetically diverse biological entities on Earth^1^. For successful infection a (bacterio)phage needs to (i) recognize and bind to a cognate receptor on the cell surface of its host, and (ii) overcome the natural barriers presented by the cell wall and membrane(s) so as to allow genome delivery into the cell cytoplasm. Several phage-encoded proteins are involved in these processes, of which the most important are the receptor binding (RBP), the tail-associated lysin (Tal) and the tail tape measure proteins (TMP) of tailed phages. The RBP specifically recognises and irreversibly attaches to the cell surface receptor molecule, which may be a carbohydrate^2^,^3^ (as present in (lipo)teichoic acids^4^, lipopolysaccharides or cell wall polysaccharides) or a membrane-associated protein^5^,^6^,^7^. Tal proteins frequently encompass peptidoglycan hydrolytic domains, which locally degrade the cell wall thereby enhancing the adsorptive abilities of the phage and/or their ability to infect cells with highly cross-linked cell walls^8^,^9^. Finally, upon irreversible attachment to the receptor, it is believed that a signal is transmitted that initiates a conformational change in the distal tail region, resulting in the ejection of the TMP, which then reconfigures into a channel to deliver the phage genome through the cell envelope into the host cell cytoplasm^10^,^11^. It was formerly assumed that TMP and Tal functions were encoded by a single protein in the well-studied coliphage T5, however, immuno-localisation assays have recently proven that the above-mentioned protein (pb2) is not located in or associated with the tail fibres, supporting the notion that pb2 constitutes the tail tube with a C-terminal hydrophobic domain at the tail tip^10^,^12^,^13^,^14^. The precise role of the TMP in the DNA injection process remains obscure and is a subject of growing interest. Significant advances have been made in the characterisation of TMP functionality for phages infecting Gram-negative bacteria such as the *Myoviridae* phage T4 and the *Siphoviridae* phages T5, HK97 and lambda^10^,^12^,^15^,^16^,^17^.

Cryo-electron tomography of *Escherichia coli* minicells with adhered phage T4 revealed that following binding of the six short tail fibres and baseplate to the receptor, transfer of genetic information is initiated by the contraction of the tail sheath and insertion of the tail tube into the cell’s outer membrane^15^. This is followed by an observed fusion between the outer and cytoplasmic membranes. This movement of the membranes towards the incoming tail tube creates an ion-permeable channel through which the DNA is translocated^15^. The TMP of T4 was also defined as being responsible for the determination of tail length, as reflected by its name^18^,^19^. Compared to the *Myoviridae* phages, members of the *Siphoviridae* family are structurally less complex, lacking the tail sheaths and long tail fibres typically associated with *Myoviridae* phages. Their tails are non-contractile and therefore the process of DNA translocation must operate via a somewhat distinct process to those of the *Myoviridae*. T5 and lambda were among the first Siphophages to be analysed with respect to their DNA injection processes. In lambda, the presence of the LamB receptor in liposomes caused a protrusion of the TMP from the distal region of the tail, which interacted directly with the liposomes forming a channel through which ions could travel^20^. This finding was consolidated by cryo-electron tomography studies following the early infection stages of the coliphages T7 and T5^21^,^22^. More recently, the genome injection process of coliphage HK97 was shown to require the inner membrane glucose transporter protein PtsG and a periplasmic chaperone (named FkpA) of the host cell^12^.

From the available information, it is clear that the TMP is a multifunctional protein with roles in tail length determination (in concert with tail assembly chaperones), connection of the capsid and distal tail regions, and genome delivery^23^,^24^. While various TMPs of Gram negative-infecting bacteria have received considerable scientific attention, those of phages infecting Gram positive bacteria have not yet enjoyed an in-depth scrutiny. Such investigations are interesting so as to understand what role TMP plays during DNA injection across the thick peptidoglycan layer of Gram positive bacteria, a formidable barrier that is absent in the Gram negative cell. In 2000, Pedersen and colleagues identified the TMP of the *Lactococcus lactis* phage TP901-1 (designated here as TMP_TP901-1_)^25^. Mutant derivatives of TMP_TP901-1_ were generated and analysed with respect to their impact on infection and morphology^25^. The removal of the N- and C-terminal regions of TMP_TP901-1_ and insertion of an amber mutation in the corresponding gene caused a reduction in the infective efficiency of the phage and a reduction in plaque size. Conversely, duplication of the N-terminal region had no significant impact on the phage titre or plaque size. Definition of the tail length-determining properties of this protein were derived from electron micrographic analysis of the N-terminal deletion and insertion mutants which revealed a 30% decrease or increase in tail length consistent with the 29% aa content removal or insertion in the mutants. Structural analysis of the tail region of the *Bacillus subtilis* phage SPP1 during the DNA ejection process revealed a reorganization in the internal wall of the tail tube following its attachment to the host, culminating in the opening of the portal vertex and in turn permitting DNA release^11^.

The current study describes a thorough bioinformatic analysis combined with characterisation of more than fifty mutants of the gene encoding TMP_TP901-1_ (*tmp*_*TP901-1*_). Through this analysis, we propose that the protein encodes (at least) twenty nine repeated sequences of 11 or 18 aa residues that influence tail length and infectivity, and two hydrophobic regions that may promote efficient binding of the chaperone and thus tail construction and assembly. This study represents the most comprehensive analysis of a TMP from a Gram positive-infecting phage, thereby generating a representative model for Siphophages that infect such bacteria.

## Results

### Characterisation of TMP_TP901-1_

**Figure 1:**
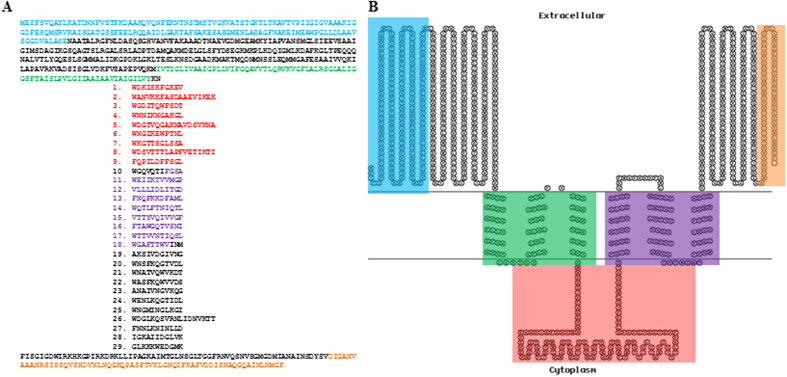
Panel A is a representation of the amino acid sequence of TMP_TP901-1_ with the 29 repeats indicated numerically and the N-terminal (light blue), C-terminal (orange), hydrophobic regions (green and purple) and cytoplasmic domain (red) highlighted by colour in the text. Panel B is a Topo2 transmembrane model of the TMP with the above-mentioned domains highlighted using the corresponding colour code to the coloured text in Panel A.

Secondary structure prediction of TMP_TP901-1_ revealed a largely α-helical content, with the exception of the C-terminal extremity of TMP_TP901-1_, which possesses a short coiled and β-sheet region (the potential significance of which is discussed below). This is congruent with the work of Katsura and Hendrix who found a similar secondary structure for lambda TMP^26^. The TMPs of several phages contain (partially) repeated regions with an 11 or 18 aa periodicity and commonly containing aromatic acids at particular positions^27^,^28^. In the lactococcal phage p2, a 40 amino acid repeat was identified, while the authors suggest that this may be further dissected to an 11 or 11-11-18 residue repeat formation using tryptophan or phenylalanine as a marker, respectively. Approximately 5% of phage-encoded TMPs or predicted TMPs examined possessed one or other of these repeat formations^27^. On this basis, manual alignment of Trp and Phe residues, spaced 11 or 18 amino acids apart, resulted in the identification of 29 proposed aromatic residue-containing repeat sequences (Fig. 1A). Four of the deduced repeat sequences measured 18 amino acids in length, with each of these repeats beginning with a Trp residue. The remaining 25 repeats of TMP_TP901-1_ are proposed to be 11 amino acids in length.

Analysis of TMP_TP901-1_ revealed the presence of two particularly hydrophobic regions that may represent membrane-associated domains, each with three putative transmembrane-spanning regions (Fig. 1B). The first hydrophobic region spans from residue I_390_ to Y_460_, the second from residues F_591_ to V_679_. In addition, the topological prediction of TMP_TP901-1_ suggests that the stretch of 129 amino acids between the two hydrophobic regions is located within the bacterial cytoplasm, while the N- and C-terminal extremities of TMP_TP901-1_ are situated extracellularly.

### Mutant construction

To elucidate the role of various TMP domains (as defined in the bioinformatic analysis described above) in virion/tail assembly and phage infectivity, a series of 43 in-frame deletion mutants of TMP_TP901-1_ was generated and their details are listed in Table 1. Additionally, a series of deletions in which repeats 1–3 plus individual amino acids of increasing magnitude covering the fourth repeat (Δ1–3.1, Δ1–3.2 etc.) were generated to understand the importance of individual amino acids within a single repeat (Table 2). Finally, two mutants inserting six and 11 alanine residues (ΔAla6 and ΔAla11, respectively) were generated to ascertain the effects of replacing the aromatic residue at the proximal end of the repeat and the remaining residues with 11 consecutive non-polar aliphatic alanine residues (Table 2). They were inserted in a background where the entire fourth TMP repeat alone (Δ4) had been deleted. Thus, a total of 56 mutants were constructed.

### Characteristics of repeat deletion mutants

*L. lactis* NZ9000 was previously lysogenised with phage TP901-1*erm* thus conferring an erythromycin-resistant phenotype on the lysogenised host^8^. In the integrated state, it was possible to generate a series of mutations in the target gene (*tmp*_*TP901-1*_) using the so-called ssDNA recombineering approach (see Materials and Methods). Induction of the (mutated) prophage(s) was achieved using mitomycin C and the resulting phage lysates were employed in plaque assays and lysogeny conversion assays on the sensitive host strain, *L. lactis* 3107. Successful infection of 3107 by TP901-1*erm* or its derivatives may have one of two outcomes: (i) visible plaques are formed on a lawn of the host or, (ii) in the absence of plaque formation, the phage may inject its DNA and integrate within the hosts’ chromosome, thus conferring erythromycin resistance. In this study, mutant derivatives of the parent phage were assessed for their ability to form plaques and to lysogenise the host as an indication that (partially) infective particles are produced (Table 1).

Twenty three mutants were specifically generated to dissect the repeat-containing region of TMP_TP901-1_ and to assess the effect of sequentially removing repeats from either/both the proximal and distal ends of the proposed repeat domains. From the proximal end, the removal of up to three repeats (Δ1–2, Δ1–3) had little or no impact on phage infectivity and lysogeny capabilities (Table 1). Similarly, the removal of five, eight, nine, twelve, fifteen and eighteen repeats from the proximal end of the repeats resulted in a relatively minor impact on infectivity, whereas the removal of ten repeats (mutant Δ1–10) caused a 4-log reduction in the efficiency of plaquing (E.O.P.; Table 1). Interestingly, mutants Δ1–4 and Δ1–7.5 were unable to form visible plaques although both were capable of lysogenising the host indicating that both derivatives remain infectious (Table 1). TMHMM^29^ modelling of the transmembrane topology of the full TMP protein and its deletion derivatives highlights that the first TMD is maintained in all repeat deletion mutants as expected. Mutant Δ1–10 is a deletion mutant for which the predicted membrane topology is altered with the first hydrophobic region unaltered as expected, while the second hydrophobic region retains just one of the three predicted membrane-spanning regions (as present in the full length protein) (Fig. S1). The substantially reduced E.O.P. and plaque size of this mutant may be a reflection of this altered membrane topology or reduced ability to efficiently recruit and bind the tail assembly chaperone.

Deletion of repeats 20/24 to 26 did not impair the functionality of the phage; however, removal of repeats 24 to 29 resulted in a 3-log reduction in E.O.P. and in combination with Δ1–9, a complete loss of plaquing ability was observed. The ability of these mutants to lysogenise the host was reduced by two logs indicating that the phages retained some residual infective activity. These findings suggest that the three terminal repeats (27 to 29 inclusive) play a role in efficient plaque formation and internalisation as the frequency of lysogenic conversion is also negatively impacted in their absence. The plaques formed by mutants Δ20–26 and Δ24–26 were comparable to those produced by the parent phage (1.5 mm clear plaques), while those produced by mutant Δ24–29 were fuzzy and reduced in diameter (Table 1). Reduced plaque size was also observed for mutants Δ1–5, 24–26; Δ1–9, 24–26 and Δ1–9, 20–26, which similarly displayed reduced E.O.P. values. It cannot be excluded that the E.O.P. reduction observed for these mutants may result from the reduced visibility of the plaques, thereby impeding an accurate count and in such situations the reduction in plaque size and clarity is an important feature of the mutants.

### Dissection of a single repeat

To understand the importance of (i) individual amino acids and (ii) the length/periodicity of a repeat element, a single repeat was genetically dissected by sequentially deleting a single amino acid from the fourth repeat in combination with repeats 1–3 (for ease of detection of the mutants). This particular repeat was selected as it was known that Δ1–3 displays similar plaque forming and lysogeny characteristics to that of the wild type phage, and because deletion of larger DNA regions (rather than individual codons) facilitated the detection of desired mutants based on size differences of the generated *tmp*_*TP901-1*_ deletions. Additionally, the effect of removing a half repeat (i.e. in the wild type background) was assessed by generating the mutant Δ3–3.5. This mutant was constructed by deleting the complete third repeat and the first five amino acids of the fourth repeat. The deletion of the third repeat is known not to have a significant impact on the infectivity of the phage (as indicated by mutant Δ1–3, Table 1) while the additional removal of the first five amino acids of the fourth repeat (Δ3–3.5, Table 2) causes the phage to be unable to produce visible plaques. Thus, 11 mutants were constructed carrying incremental deletions and named Δ1–3.1 to Δ1–4 (=Δ1–3.11), and each mutant was characterised with respect to lysogeny and plaque forming ability. All mutants lysogenised the host at apparently equal efficiency; however, only mutants Δ1–3.3, Δ1–3.7 and Δ1–3.10 were capable of forming plaques (or at least countable plaques). Additionally, the plaque morphology of plaque-forming mutants was altered as they were reduced in diameter from 1.5 mm (WT and mutant Δ1–3) to 0.75 mm (mutant Δ1–3.3) and 0.5 mm (mutants Δ1–3.7 and Δ1–3.10) (Table 2).

**Figure 2:**
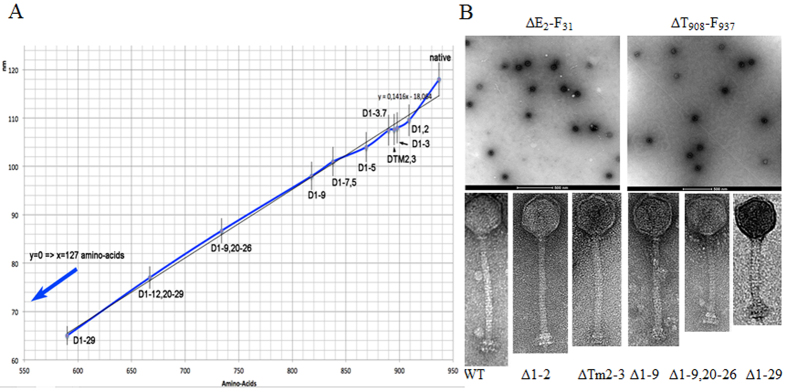
Panel A is a graph depicting the reduction in tail length of the indicated mutants derived through electron microscopic analysis. Panel B highlights representative micrographs of the indicated mutants. On the top the removal of thirty amino acids from the N- and C-termini, respectively cause aberrant phage assembly while the removal of repeats and hydrophobic regions (bottom) exhibit sequential tail length reduction as an increasing number of amino acids are removed.

Morphological analysis of purified preparations of these mutants by electron microscopy revealed that intact particles were produced by Δ1–3.3, Δ1–3.7 and Δ1–3.10, as expected since they produce plaques similar to the wild type. Similarly, Δ1–3.4, Δ1–3.9 and Δ1–4 (Δ1–3.11) produced intact particles despite not being capable of plaque formation (Table 2). Interestingly, the remainder of the mutants (Δ1–3.1, Δ1–3.2, Δ1–3.5, Δ1–3.6, Δ1–3.8 and Δ3–3.5) appeared as heads only, or as separated head and tail structures (Fig. 2). The effect of pH and freeze-thaw treatments on the stability of these mutants was explored in an effort to define if the storage and handling conditions would impact on their integrity. However, no significant loss of infectivity was observed under the assessed conditions (data not shown). Removal of the fourth repeat (Δ4) (in a native protein background) and its replacement with six and 11 Alanine residues, respectively, had little impact on the above-mentioned infection characteristics. The results suggest that the helical periodicity of the tail tube structure bears greater significance than the nature of the side-chain residues within the repeated elements of the protein (at least in the case where only one repeat is replaced).

### Characterisation of deletion mutants in hydrophobic regions

*In silico* analysis of the TMP of TP901-1 identified two predicted hydrophobic regions, each with three putative membrane-spanning regions (Tm). In this study, we aimed to explore the possibility that these hydrophobic regions constitute transmembrane domains (TMDs) involved in pore formation through the membrane to facilitate DNA injection. In the case that these hydrophobic regions represent transmembrane domains, their removal would thus be expected to negatively affect the orientation and insertion of the TMP in the bacterial cell membrane and thus the infectivity of the phage. ΔTm1-3 and ΔTm1-6, which represent mutants lacking the first and both hydrophobic regions, respectively, were incapable of forming plaques or lysogenising the host, highlighting their essential nature. Mutants ΔTm3 and ΔTm2-3, which lack the named predicted membrane-spanning regions (numbered 1–6), were incapable of plaque formation, while lysogenic conversion was possible, although to a very limited extent in the case of ΔTm2-3. Additionally, the removal of Tm1 in addition to Tm2-3 (mutant ΔTm1-3), completely obliterates viable infection by the phage indicating that this membrane-spanning region is essential to the development of an infective virion. Conversely, mutants ΔTm4 and ΔTm4-6 were shown to behave as the wild type phage, indicating that the second hydrophobic region is not essential for infection. Furthermore, electron microscopic analysis of ΔTm2-3 (Fig. 2B) identified that this mutant forms intact particles, while ΔTm1-6 forms separated heads and tails (data not shown), highlighting the instability of this mutant or its inability to assemble correctly. Therefore, while it may be concluded that the hydrophobic regions may be beneficial for chaperone recruitment during tail construction and/or virion assembly^28^, their role in membrane insertion and DNA translocation seems less likely since the second hydrophobic region has little impact on the infectivity of the phage.

### Characteristics of N- and C-terminal deletion mutants

It was hypothesized that the N-terminus of the TMP is involved in interactions with the head-tail connecting region of the phage, while the C-terminus is proposed to be involved in interactions with the other components of the so-called initiator complex (Dit and Tal) at the distal tail region, which forms the hub around which the baseplate is constructed. To evaluate these assumptions, mutants lacking the N- and C-terminal 30 aa residues were generated (ΔE_2_-F_31_ and ΔT_908_-F_937_, respectively). Neither mutant formed plaques nor could they lysogenise the host, thereby supporting their presumed essential role in attaching the tail to the respective features at either end of the tail. While the distal ends of the TMP are clearly critical to developing intact and functioning phage particles, it was unclear if the construction of an intact particle is dependent on protein domains beyond the distal reaches of the protein. To this end, ΔF_31_-I_61_, ΔI_62_-L_141_, ΔF_31_-L_141_ and ΔA_142_-E_154_ were generated to assess the effect of N-terminal deletions on the infective function of the phage. Each of these deletions leads to complete loss of infectivity with neither plaque formation nor lysogenisation of the host detectable. In contrast to this, deletions at the C-terminal end from aa residues 810 to 875 maintain their ability to infect and lysogenise the host albeit to a limited extent (Table 1). Furthermore, ΔF_810_-G_842_ and ΔF_810_-V_875_ in combination with Δ1–29 (the entire proposed repeat unit) causes a loss of plaque forming ability and a further reduction in the frequency of lysogeny. A subsequent deletion mutant additionally lacking aa residues 876–908 (Δ1-29, F_810_-T_908_) is unable to form plaques or lysogenise the host. Electron microscopic analysis of Δ1–29, F_810_-T_908_ indicates that this mutant lacks tails, thus impeding its infectious activity (data not shown).

### Assessment of tail length

The deletion mutants Δ1–2; Δ1–7.5; Δ1–9; Δ1–9, 20–26, Δ1–12, 20–29 and Δ1–29 displayed increasingly shorter tail lengths of 109.5, 101, 98, 86.7, 77 and 65 nm, respectively, compared to the 118 nm tail of the parent phage, as determined in this study (and previous studies)^30^. Additionally, the tail length of ΔTm2-3 was determined to be 107.6 nm. The region encoding Tm2-3 directly precedes the proposed repeat region and thus apparently contributes to tail length determination in addition to the proposed repeat region. When the virion’s tail length was plotted against TMP size (as expressed in amino acid number) an inverse correlation was observed. Extrapolation of the line to a tail length of 0 nm crosses the axis at a value of 127 amino acids. This value would theoretically represent a TMP of a virion without a tail. In other words, it may account for the N- and C-terminal domains, which is presumed to be embedded in the capsid-tail connector/associated with the tail terminator and the Dit-Tal structures, respectively. Indeed, the respective dimensions of the individual domains remain unknown. This scheme also indicates that ~810 amino acids of the TMP contribute to tail formation. Dividing the tail’s length (118 nm) by this number yields a value of 0.145 nm per amino acid, consistent with the estimation by Katsura and colleagues (0.15 nm)^26^. Noteworthy, the average length of an amino acid in an extended alpha-helix is 1.47 Å. Here, 810 amino acids in an extended alpha-helix would yield a TMP length within the tail of 1190 Å, almost identical to the TP901-1 tail length of 1180 Å. This implies that the TMP is straight and fully extended in the tail.

### Evaluation of tail production in non-infective phage mutants

In order to define if non-plaque forming mutants exhibit this trait as a consequence of the absence or instability of tailed structures or impaired assembly, complementation assays were performed. To this end, intact tails (produced from a mutant that does not produce the major head protein^31^) were added exogenously to the crude lysate of non-plaque forming mutants and in all cases, (almost) full infectious activity was restored in terms of lysogeny conversion at levels comparable to that of the parent phage (Table 1).

## Discussion

The phage tail is a multifunctional structure that acts as a connector between the phage head and the host-recognition device, and is comprised of a channel for DNA translocation into the host cell. A set of 56 TP901-1*erm*-derived mutants was generated, each carrying a mutated *tmp*_TP901-1_ gene, aimed at performing an in depth analysis and dissection of this protein, which is an integral part of the tail tube.

In this study, 29 proposed repeat elements were identified based on the identification of aromatic residues at 11 or 18 residue intervals (Fig. 1). Removal of the entire repeat unit (Δ1–29), which lacks 347 aa residues compared to the TMP of the parent phage prevents plaque formation, but retains the ability to lysogenise the host. Furthermore, a direct correlation between deletion size and (reduced) tail length was observed by electron microscopy for deletions both in the repeat region and also in the first hydrophobic region (Fig. 2). The presence of such repeats in TMPs of phages infecting a number of bacterial genera has been defined (approximately 5% of phage proteins assessed)^27^ and furthermore, the presence of complementary aromatic residues at a similar periodicity in the TMP chaperone of the 936 phage p2 makes it tempting to speculate that these two elements interact via these complementary regions to stabilise the tail assembly process^28^. However, while these aromatic residues are markers of the proposed repeats, they do not appear to be essential since the replacement of an entire 11 aa repeat with alanine residues bears no impact on the infectivity of the phage (Table 2). These results indicate that the repeat region alone is not responsible for tail length determination, although the presence of repeats is perhaps a natural facilitator of domain duplication/deletion to incorporate flexibility into the structure such that it may adapt to emerging hosts and environmental conditions. Such repeat elements are not observed in all phage TMPs, therefore, their potential role in facilitating adaptive responses in terms of tail length to accommodate the host and environment may be an advantageous rather than essential feature of phage TMPs.

It is possible that a long tail/TMP may be beneficial in positioning the phage for optimal infection and possibly aiding movement through the surface polysaccharide and thick peptidoglycan layers culminating in the docking of the phage (tail tube) at the cell membrane in a more efficient manner than those with shorter tails depending on the depth of the outer surface structures of the host. There is currently no structure available for phage TMPs. Recently, the cryoEM atomic structure of phage T4 baseplate and tail at atomic resolution revealed the 3D structure of all its components except the TMP that was disordered in the tail channel^17^. According to the size of the cavity and of the EM density, the TMP within the tail tube was predicted to exist as a hexamer, in agreement with the 6-fold symmetry of the tail^30^,^32^.

Concerning the N- and C-termini of the TMP in siphophages, we can deduce some information from low resolution EM structures. In a recent paper, the structure of an SPP1 complex including the portal, the connector, the tail terminator and the MTP was reported at medium resolution (7 Å)^33^. In this structure, the EM density of the TMP N-terminus is visible, although disordered. The volume of the TMP putatively interacting with the connector and tail terminator was found to be quite small. In contrast, complete EM structures of siphophages p2 and TP901-1^32^,^34^ revealed that the baseplate interior, formed by the cavity of the Dit-Tal complex, is quite large, and filled by an EM density assigned to the TMP C-terminus.

In the current study, electron microscopic analysis revealed that deletion of the N- or C-terminal 30 aa residues results in aberrant particle assembly (or lack of tail production/assembly) as only phage heads are observed (Fig. 2). Electron microscopic analysis performed in this study would suggest that the C-terminus of the protein is essential in establishing the tail construction process as part of the initiator complex thereby substantiating the notion that the C-terminus is indeed involved in interactions with other initiator complex and/or baseplate proteins, as seen in EM structures of the virion, while the N-terminus is likely involved in attachment to the tail terminator and/or the head-tail connector protein(s).

TMP_TP901-1_ is predicted to contain two hydrophobic regions which were examined to assess the possibility that they may form transmembrane domains. This study proved the essential nature of the first hydrophobic region as its removal obliterated the phage’s ability to form plaques or lysogenise the host while deletion of the second hydrophobic region appeared to exert minimal impact on these infection characteristics (Table 1). Since both hydrophobic regions do not exert an impact on infectivity of the phage it is impossible to assert that these domains are essential to membrane insertion in the host. Since the hydrophobic stretches of TMPs have previously been affiliated with chaperone binding^28^, it is possible that these TMDs are required for efficient tail construction and assembly through the optimal recruitment of the chaperone.

## Conclusions

Employing a combined approach of computational and genetic analysis of TMP_TP901-1_, it was possible to predict and characterise two hydrophobic regions and twenty nine repeat sequences. Based on the findings of this study, we conclude that the main prerequisites to a fully functioning TMP in phage TP901-1 are (i) the presence of the first hydrophobic region; (ii) the maintenance of at least 154 aa residues at the N-terminus and approximately 60 aa at the C-terminus; (iii) repeats 19–29 that form part of the predicted extracellular C-terminal domain. The latter prerequisite is only essential for plaque formation while lysogeny does not require this feature indicating that an intact and infectious particle is still produced in the absence of the repeat region. The repeated sequence region accounts for only 347 of the proposed 810 tail-length determining residues (Fig. 2) and its function is likely in providing flexibility in the gain/loss of repeat sequences to facilitate adaptation to emerging hosts. The presence of repeat sequence elements in the TMPs of a number of tailed phages highlights the evolutionary conservation of such beneficial features in the phage realm. Given the importance of tailed phages in both ecological balancing and the evolution of their host bacteria, it is imperative that we develop a detailed understanding of the phage infection process. This study represents the most thorough molecular analysis of the TMP of a Gram positive-infecting phage and has provided essential data towards understanding the *modus operandi* of TMPs of tailed bacteriophages, particularly those of Gram positive bacteria.

## Materials & Methods

### In silico analysis of TMP_TP901-1_

The Genbank accession number for TP901-1 is NC_002747. The sequence of TMP_TP901-1_, corresponding to the product of ORF45, was manipulated using DNASTAR software package (Version 7.2, 2007; DNASTAR Inc., Madison, WI). Repeat sequences in TMP_TP901-1_ were deduced by manually aligning Trp and Phe residues with 11 or 18 amino acid periodicity. Hydrophobic and predicted transmembrane domains were identified using TMHMM^35^, while protein transmembrane topology was characterised using TOPO2^36^. The secondary structure of TMP_TP901-1_ was predicted using SABLE^37^ and alignment of TMP amino acid sequences was performed using ClustalW^38^.

### Bacterial strains, phages and growth conditions

Bacterial strains and phages used in this study are listed in Table 1. Lactococcal strains were grown at 30 °C without agitation in M17 broth (Oxoid Ltd., UK) supplemented with 0.5% glucose. Phage lysates for the various biological characterisation assays were induced from (at least) three separate overnights of NZ9000-Cro_t712_ lysogenized with TP901-1*erm*, or a particular mutant derivative using 0.5 μg/ml mitomycin C when the growing cultures reached an optical density at 600 nm (OD600) of approximately 0.2. Polyethylene glycol 8000 (PEG 8000) precipitation of phage particles (or component parts) of the TP901-1*erm* phage or a particular mutant derivative, and their subsequent purification, was performed as described previously^31^.

### Mutant generation and screening

Recombineering mutagenesis was performed as described previously^8^,^39^,^40^, with the following adjustments. Briefly, recombineering oligonucleotides designed to delete regions of *tmp*_*TP901-1*_, which are listed in Supplementary Table S1, bound to approximately 40–45 nucleotides of TP901-1*erm* genomic DNA flanking each deletion. Genomic deletions created in this study were on average designed to be approximately 100 nucleotides, although sequences as short as 39 nucleotide bases (TMP mutant ΔA_142_-E_154_) or as long as 240 bases (mutant ΔI_62_-L_141_) were successfully removed in a single recombineering experiment. Following transformations involving recombineering oligonucleotides, cells were allowed to recover for approximately 30–45 minutes before spread-plating the necessary dilutions on GM17 agar plates supplemented with 5 μg/ml erythromycin. Screening was performed by PCR amplification using NZ9000-Cro_t712_-TP901-1*erm* colonies, or derivatives thereof carrying the specific introduced mutations, as template DNA. The integrity of the sequence of each introduced mutation in *tmp*_*TP901-1*_ was confirmed by Sanger sequencing the PCR product generated during screening (MWG Eurofins, Germany).

### Phage assays

Plaque assays of TP901-1*erm* and TMP_TP901-1_ mutant derivatives were performed using standard protocols^41^. The efficiency of plaquing (EOP) of TP901-1*erm* mutants, compared to the wild-type control, was calculated by dividing the plaque-forming units per millilitre (pfu/ml) of the particular TP901-1*erm* mutant by the pfu/ml of the wild type TP901-1*erm* control. EOP assays were performed on three separate replicates, and the results averaged. The plaque size and morphology were also noted to define any aberrations in plaque formation.

Frequency of lysogeny experiments were performed as described previously employing filtered crude lysates following mitomycin C-induction of TP901-1*erm* and its TMP_TP901-1_ mutant derivatives^8^,^42^. The ability to measure frequency of lysogeny is based on bacterial acquisition of the adenine methylase-encoding gene present in the TP901-1*erm* phage genome or its mutant derivatives, conferring resistance to 2.5 μg/ml erythromycin (Erm2.5)^43^,^44^. All results presented are the average of at least triplicate experiments and a positive control of the wild type TP901-1*erm* was included in all assays as a comparator for the frequency of lysogeny, which was calculated by dividing the number of Erm-resistant cfu.ml^−1^ by the total population (cfu.ml^−1^) counted on GM17 agar without antibiotic selection.

The impact of environmental factors on the stability of selected mutants was assessed using caesium chloride purified phage preparations of the wild type phage and its mutated derivatives Δ1–2, Δ1–3.1, Δ1–3.2, Δ1–3.5 and Δ3–3.5. For pH sensitivity testing, 0.2 ml of the purified phage sample was suspended in 0.8 ml GM17 broth adjusted to pH 4 or at pH 6.5 (standard broth as a control). Thermal sensitivity was assessed by storing the purified samples at −20 °C for 1 hour followed by a 20 minute thaw period and a second freezing step for 20 minutes before the sample was finally thawed and tested for lysogenic conversion as described above and using a multiplicity of infection (MOI) of 0.5. All assays were performed in triplicate and the presented results are the average of these data.

### Electron microscopic analysis of phages

For negative staining, 5 μl of each sample were applied onto glow-discharged carbon-coated grids (Agar Scientific, Stansted, UK) and incubated for 1 min. The grids were washed with 5 μl of deionized water before incubating for 30 sec in 1% (w/v) of uranyl acetate (Agar Scientific, Stansted, UK.). CCD images were collected using a Tecnai Spirit operated at 120 Kv and a 2 K × 2 K CCD camera. The tail length measurements were obtained by averaging the single tail lengths from ensembles of 10 to 18 single mutated virions images at the same scale.

## Additional Information

**How to cite this article**: Mahony, J. *et al*. Functional and structural dissection of the tape measure protein of lactococcal phage TP901-1. *Sci. Rep.* **6**, 36667; doi: 10.1038/srep36667 (2016).

## Supplementary Material

Supplementary Information

## Figures and Tables

**Table 1:** Characteristics of TMP-mutant derivatives of TP901-1.

TP901-1 mutant	#aa deleted	Tail length (nm)	E.O.P.	Plaque morphology	^γ^Frequency of lysogeny (FL)	FL upon addition of tails
TP901-1*erm* WT	0	118	1	Clear, 1.5 mm	4.4 × 10^−3^	4.8 × 10^−3^
Δ1–2	29	109.5	4.5	Clear, 1.5 mm	3.8 × 10^−3^	ND
Δ1–3	40		6.8 × 10^−2^	0.75 mm	1.4 × 10^−3^	ND
Δ1–4	51		≤1.5 × 10^−8^	NA	9.0 × 10^−5^	ND
Δ1–5	69		3	Clear, 1 mm	1.8 × 10^−3^	ND
Δ1–7.5	100	101	≤1.5 × 10^−8^	NA	1.1 × 10^−4^	2.3 × 10^−3^
Δ1–8	109		3	0.75 mm	5.3 × 10^−3^	ND
Δ1–9	120	98	0.88	Clear, 1 mm	4.5 × 10^−3^	ND
Δ1–10	131		6.8 × 10^−4^	0.5 mm	3.5 × 10^−3^	ND
Δ1–12	153		6	Clear, 1.5 mm	1.9 × 10^−3^	ND
Δ1–15	186		5.4	Clear, 1.5 mm	2.6 × 10^−3^	ND
Δ1–18	219		1.4	Clear, 1.5 mm	7.6 × 10^−3^	ND
Δ1–29	347	65	≤1.5 × 10^−8^	NA	1.6 × 10^−5^	6.5 × 10^−4^
Δ20–26	84		6.5	Clear, 1.5 mm	2.2 × 10^−3^	ND
Δ24–26	40		4.3	Clear, 1.5 mm	2.4 × 10^−3^	ND
Δ24–29	73		2.0 × 10^−3^	Fuzzy, 1 mm	3.5 × 10^−3^	ND
Δ1–5, 24–26	109		0.6	0.5 mm	4.2 × 10^−3^	ND
Δ1–9, 24–26	160		8.2 × 10^−3^	0.5 mm	1.7 × 10^−3^	ND
Δ1–9, 20–26	204	86.7	0.34	0.5 mm	2.4 × 10^−3^	ND
Δ1–9, 24–29	193		≤1.5 × 10^−8^	NA	4.3 × 10^−5^	3.5 × 10^−3^
Δ1–9, 20−29	237		≤1.5 × 10^−8^	NA	1 × 10^−4^	2.1 × 10^−3^
Δ1–12, 20–29	270	77	≤1.5 × 10^−8^	NA	4.6 × 10^−4^	1.0 × 10^−3^
Δ1–15, 20–29	303		≤1.5× 10^−8^	NA	3.4 × 10^−4^	1.5 × 10^−3^
Δ1–18, 20–29	336		≤1.5× 10^−8^	NA	3.4 × 10^−4^	ND
ΔTM1-3	71		≤1.5 × 10^−8^	NA	≤1.5 × 10^−8^	2.8 × 10^−3^
ΔTM3	19		≤1.5 × 10^−8^	NA	3.2 × 10^−4^	2.0 × 10^−3^
ΔTM2-3	43	107.6	≤1.5 × 10^−8^	NA	1.3 × 10^−7^	2.6 × 10^−3^
ΔTM4-6	89		6.8 × 10^−2^	NA	4.4 × 10^−3^	ND
ΔTM1-6	160	No tails	≤1.5 × 10^−8^	NA	≤1.5 × 10^−8^	4.0 × 10^−3^
ΔTM4	23		3.4	Clear, 1.5 mm	8.9 × 10^−4^	ND
ΔE_2_-F_31_	30	No tails	≤1.5 × 10^−8^	NA	≤1.5 × 10^−8^	3.1 × 10^−3^
ΔT_908_-F_937_	30	No tails	≤1.5× 10^−8^	Clear, 1.5 mm	≤1.5 × 10^−8^	2.7 × 10^−3^
ΔF_810_-G_842_	33		9.5 × 10^−7^	pinpoint	4.4 × 10^−4^	ND
ΔL_843_ –V_875_	33		1.4 × 10^−5^	pinpoint	7.4 × 10^−5^	ND
ΔF_810_-V_875_	66		2.0 × 10^−6^	pinpoint	5.4 × 10^−5^	ND
Δ1–29, F_810_-G_842_	380		≤1.5 × 10^−8^	NA	4.1 × 10^−5^	2.8 × 10^−3^
Δ1–29, F_810_-V_875_	413		≤1.5 × 10^−8^	NA	3.4 × 10^−6^	1.9 × 10^−3^
Δ1–29, F_810_-T_908_	446	No tails	≤1.5 × 10^−8^	NA	≤1.5 × 10^−8^	3.5 × 10^−3^
Δ F_31_-I_61_	30		≤1.5× 10^−8^	NA	≤1.5 × 10^−8^	6.0 × 10^−4^
Δ F_31_-L_141_	110	No tails	≤1.5 × 10^−8^	NA	≤1.5 × 10^−8^	5.8 × 10^−4^
Δ I_62_-L_141_	80		≤1.5 × 10^−8^	NA	≤1.5 × 10^−8^	3.4 × 10^−3^
ΔA_142_-E_154_	13		≤1.5 × 10^−8^	NA	≤1.5 × 10^−8^	2.2 × 10^−3^
Δ I_62_- L_141,_ Δ1–29, F_810_-T_908_	526		≤1.5 × 10^−8^	NA	≤1.5 × 10^−8^	2.6 × 10^−3^

All results are the average of at least triplicate assays. ND = Not determined; NA = Not applicable as no plaques were formed. E.O.P. = efficiency of plaquing relative to the parent phage. Tail length of selected mutants examined by electron microscopy. ^γ^Frequency of lysogeny is represented as the number of erythromycin-resistant colonies as a proportion of the total population of cells. For mutants with impaired lysogenic abilities, phage tails were added exogenously to assess the production of intact capsids and subsequent assembly with the provided tails thereby producing an infective particle.

**Table 2:** Characteristics of TMP mutants of the fourth repeat.

TP901-1 mutant	#aa deleted	Phage morphology	E.O.P.	FL of phage
TP901-1*erm* WT	0	Intact	1	4.6 × 10^−4^
Δ1–3.1	41	Heads only	≤3.0 × 10^−7^	2.5 × 10^−4^
Δ1–3.2	42	Heads only	≤3.0 × 10^−7^	6.5 × 10^−4^
Δ1–3.3	43	Intact	4.0^*^	2.4× 10^−3^
Δ1–3.4	44	Intact	≤3.0 × 10^−7^	2.1 × 10^−4^
Δ1–3.5	45	Heads only	≤3.0 × 10^−7^	8.8 × 10^−4^
Δ1–3.6	46	Heads only	≤3.0 × 10^−7^	6.5 × 10^−4^
Δ1–3.7	47	Intact	6.8^*^	6.7 × 10^−4^
Δ1–3.8	48	Heads only	≤3.0 × 10^−7^	5.5 × 10^−4^
Δ1–3.9	49	Intact	≤3.0 × 10^−7^	6.3 × 10^−4^
Δ1–3.10	50	Intact	0.8^*^	5.4 × 10^−4^
Δ1–3.11	51	Intact	≤3.0 × 10^−7^	9.0 × 10^−5^
Δ4::Ala6	NA	ND	0.08	1.7 × 10^−4^
Δ4::Ala11	NA	ND	0.06	1.5 × 10^−4^
Δ3–3.5	16	Heads, long tails	≤3.0 × 10^−7^	7.3 × 10^−4^

*Denotes reduced plaque size in mutants capable of plaque formation. FL = Frequency of lysogenic conversion.
