# Supplementary material for: Functional and structural dissection of the tape measure protein of lactococcal phage TP901-1
Source: Sci Rep. 2016 Nov 8;6:36667. doi: 10.1038/srep36667 (PMC5099701; doi:10.1038/srep36667)
Supplement: Supplementary Information [file srep36667-s1.pdf]

Supplementary information

Functional and structural dissection of the tape measure protein of lactococcal phage TP901-1

Jennifer Mahony, Mona Alqarni, Stephen Stockdale, Silvia Spinelli, Marine Feyereisen, Christian Cambillau and Douwe van Sinderen*[1,2]

Supplementary Table S1. List of primers used to generate the recombineered mutants within $tmp_{TP901\text{-}1}$

Oligonucleotide name	**Mutational background**	**Oligonucleotide sequence (5' → 3')**
Recombineering primers		
Δ1-2	wt	a*a*t*g*t*tgttccaggtatcagagaaccattgggtaatatctccccaatttttataaactaaaattcctatagctataacagccgctatggc
Δ1-5	Δ1-2	c*g*t*a*c*ctttccaaagattagtgaaccactccttaatgccattccaatttttataaactaaaattcctatagctataacagccgctatggc
Δ1-7.5	Δ1-5	a*t*c*a*a*gaattggttgaaagattgttttgattgtctcaacaaatggatttttataaactaaaattcctatagctataacagccgctatggc
Δ1-8	Δ1-5	g*a*c*t*t*gcccccataatccgctaaagaaatcaagaattggttgaaaatttttataaactaaaattcctatagctataacagccgctatggc
Δ1-9	Δ1-5	t*c*t*c*c*caagctgaaccaaagatagtttggacttgcccccaatttttataaactaaaattcctatagctataacagccgct
Δ1-10	Δ1-8	g*a*g*t*a*acaaaactggtcccataacaaccgtcttaataatctcccaatttttataaactaaaattcctatagctataacagccgctatggc
Δ1-12	Δ1-9	t*a*a*t*g*tttgccagagcatcgcaaaatctttttgaattggttaaaatttttataaactaaaattcctatagctataacagccgctatggc
Δ1-15	Δ1-12	a*a*c*t*g*tcgtccagatattagaaacagtttgaccccaagcggtaaaatttttataaactaaaattcctatagctataacagccgctatggc
Δ1-18	Δ1-15	c*c*c*g*c*t aatgaaaccattaacaattccgtcaacaatagacttggcatttttataaactaaaattcctatagctataacagccgctatggc
Δ1-29	Δ1-15, 20-29	t*g*g*c*c*ctttatgcttccgaatccaatcgccaatcccgctaatgaaatttttataaactaaaattcctatagctataacagccgctatggc
Δ24-26	WT	t*a*c*c*a*atatctagcaagtttatatttttagattgttaaaaccttgtttgactccattcacaatagcattggcagaatca
Δ20-26	Δ24-26	g*g*c*t*t*taccaatatctagcaagtttatatttttagattgttaaaaccattaacaattccgtcaacaatagacttggccatattaatgac
Δ24-29	Δ24-26	t*g*g*c*c*ctttatgcttccgaatccaatcgccaatcccgctaatgaaaccttgtttgactccattcacaatagcattggcagaatcaacgac
Δ1-5, 24-26	Δ1-5	As for Δ24-26
Δ1-9, 24-26	Δ1-5, 24-26	As for Δ1-9
Δ1-9, 20-26	Δ1-9, 24-26	As for Δ20-26
Δ1-9, 24-29	Δ1-9, 24-26	As for Δ24-29
Δ1-9, 20-29	Δ1-9, 20-26	t*g*g*c*c*ctttatgcttccgaatccaatcgccaatcccgctaatgaaaccattaacaattccgtcaacaatagacttggccatattaatgac
Δ1-12, 20-29	Δ1-9, 20-29	as for Δ1-12
Δ1-15, 20-29	Δ1-12, 20-29	as for Δ1-15
Δ1-18, 20-29	Δ1-15, 20-29	as for Δ1-18
ΔTm1-3	ΔTm2-3	t*g*c*c*c*atacttctttcccgaatttagaaattttatcccaatttttcatttttgtacaggctcaggagcagaaacaaatttatcaactaa
ΔTm3	WT	a*t*a*c*t*tctttcccgaatttagaaattttatcccaatttttggtaaagaaatagcagtaaaacttcctcctattagagca
ΔTm2-3	ΔTm3	t*g*c*c*c*atacttctttcccgaatttagaaattttatcccaatttttactctttgtagagtaacaacagcttgcccaaatattactaataa
ΔTm4	WT	g*c*a*t*c*gcaaaatctttttgaattggttaaagtccccagtgatagtttggacttgcccccataatccgctaaagaaatca

ΔTm4-6	WT	c*c*a*a*c*cattaacaattccgtcaacaatagacttggccatattaatgatagtttggacttgcccccataatccgctaaagaaatcaagaat
ΔTm1-6	ΔTm1-3	As for ΔTm4-6
ΔE_{2}-F_{31}	WT	c*t*t*t*c*cctactgtagacatcgtactattagtgttcttttccatattttcctcctttcctagttatttgctttttcatga
ΔF_{31}-I_{61}	WT	t*t*c*a*a*aatcaccacctatttttgcggctgcaaccccaattcctatgttttgaacttgtttagcagcgtctttaaatgtactaacaaaatt
ΔF_{31}-L_{141}	ΔI_{62}-L_{141}	t*t*c*a*a*aatcaccacctatttttgcggctgcaaccccaattcctatgttttgaacttgtttagcagcgtctttaaatgtactaacaaaatt
ΔI_{62}-L_{141}	WT	a*g*c*a*t*tttcagaagccaacgcaacatcaccaccagaaacagcagcgttttgaacttgtttagcagcgtctttaaatgtactaacaaaatt
ΔA_{142}-E_{154}	WT	t*t*g*a*c*tagcatcaagattaaatcctcttaaagcggtagcagcatttaagtctaaaagccccggcattgcttccattatttctttggcgtt
ΔF_{810}-G_{842}	WT	g*t*t*a*g*attgaacgttacggaagcctccagttaaaccagaattcaacttcatcccatcttcccactttttcttcaggccttttacaagtcc
ΔL_{843} –V_{875}	WT	a*c*t*a*c*tgattgagcgattagcagccgcaacatttgccccaatatcacctgtcataatggctttaccagcaggaattaaaagttttctatc
ΔF_{810}-V_{875}	ΔF_{810}-G_{842}	a*c*t*a*c*tgattgagcgattagcagccgcaacatttgccccaatatccttcatcccatcttcccactttttcttcaggccttttacaagtcc
Δ1-29, F_{810}-G_{842}	Δ1-29	g*t*t*a*g*attgaacgttacggaagcctccagttaaaccagaattcaaatttttataaactaaaattcctatagctataacagccgctatggc
Δ1-29, F_{810}-V_{875}	Δ1-29, F_{810}-G_{842}	g*t*t*a*g*attgaacgttacggaagcctccagttaaaccagaattcaaatttttataaactaaaattcctatagctataacagccgctatggc
Δ1-29, F_{810}-T_{908}	Δ1-29, F_{810}-V_{875}	a*a*t*g*t*catccacaaaggctttaaagatttgattcccaagcttcacatttttataaactaaaattcctatagctataacagccgctatggc
Δ I_{62}- $L_{141,}$ Δ1-29, F_{810}-T_{908}	Δ1-29, F_{810}-T_{908}	As for Δ I_{62}- L_{141}
ΔT_{908}-F_{937}	WT	g*t*t*t*t*gtcgtatctctaaacttgtacatttctacctcctagaatgaagccggctgttttccttggttaagattcacatca
Δ1-3	Δ1-2	t*g*t*t*c*catcccagagccctttagctccatttttaatgttgttccaatttttataaactaaaattcctatagctataacagccgctatggc
Δ1-3.1	Δ1-2	g*a*c*t*g*ttccatcccagagccctttagctccatttttaatgttgttatttttataaactaaaattcctatagctataacagccgctatggc
Δ1-3.2	Δ1-2	t*t*g*g*a*ctgttccatcccagagccctttagctccatttttaatgttatttttataaactaaaattcctatagctataacagccgctatggc
Δ1-3.3	Δ1-2	a*c*c*t*t*ggactgttccatcccagagccctttagctccatttttaatatttttataaactaaaattcctatagctataacagccgctatggc
Δ1-3.4	Δ1-2	g*g*c*a*c*cttggactgttccatcccagagccctttagctccatttttatttttataaactaaaattcctatagctataacagccgctatggc
Δ1-3.5	Δ1-2	t*t*t*g*g*caccttggactgttccatcccagagccctttagctccattatttttataaactaaaattcctatagctataacagccgctatggc
Δ3-3.5	WT	t*t*t*g*g*caccttggactgttccatcccagagccctttagctccattatttttataaactaaaattcctatagctataacagccgctatggc
Δ1-3.6	Δ1-2	a*t*t*t*t*tggcaccttggactgttccatcccagagccctttagctccatttttataaactaaaattcctatagctataacagccgctatggc
Δ1-3.7	Δ1-2	g*g*c*a*t*ttttggcaccttggactgttccatcccagagccctttagcatttttataaactaaaattcctatagctataacagccgctatggc
Δ1-3.8	Δ1-2	a*a*c*g*g*catttttggcaccttggactgttccatcccagagccctttatttttataaactaaaattcctatagctataacagccgctatggc
Δ1-3.9	Δ1-2	a*t*c*a*a*cggcatttttggcaccttggactgttccatcccagagcccatttttataaactaaaattcctatagctataacagccgctatggc
Δ1-3.10	Δ1-2	a*c*t*a*t*caacggcatttttggcaccttggactgttccatcccagagatttttataaactaaaattcctatagctataacagccgctatggc
Δ1-4	Δ1-2	a*a*c*a*c*tatcaacggcatttttggcaccttggactgttccatcccaatttttataaactaaaattcctatagctataacagccgctatggc
Δ4	WT	a*a*c*a*c*tatcaacggcatttttggcaccttggactgttccatcccaggtatcagagaaccattgggtaatatctcc ccaatttttataaac
Ala6	Δ4	a*t*t*t*t*tggcaccttggactgttccatcccatgcggcagccgctgcggcggtatcagagaaccattgggtaatatctcccca
Ala11	ala6	c*c*t*t*g*gactgttccatcccatgcggcagccgctgcggcagctgcagctgcagcggtatcagagaaccattgggtaatatct

*Phosphorothioate linkages of recombineering oligos.

Fig. S1

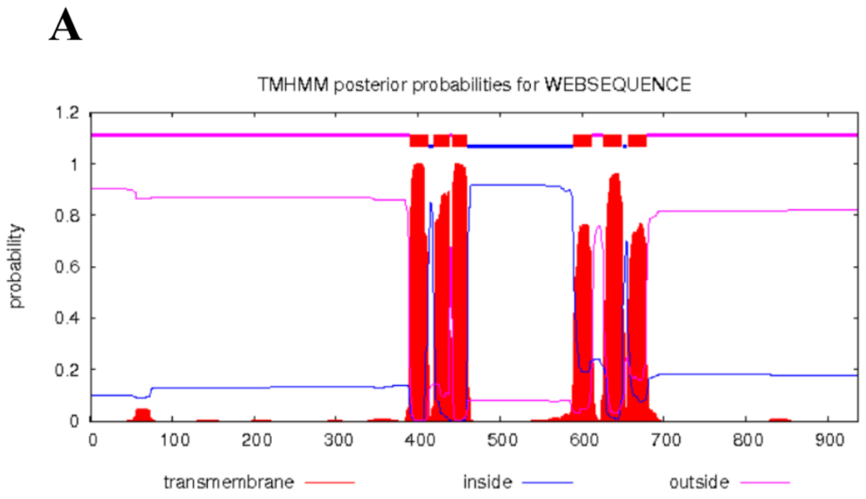

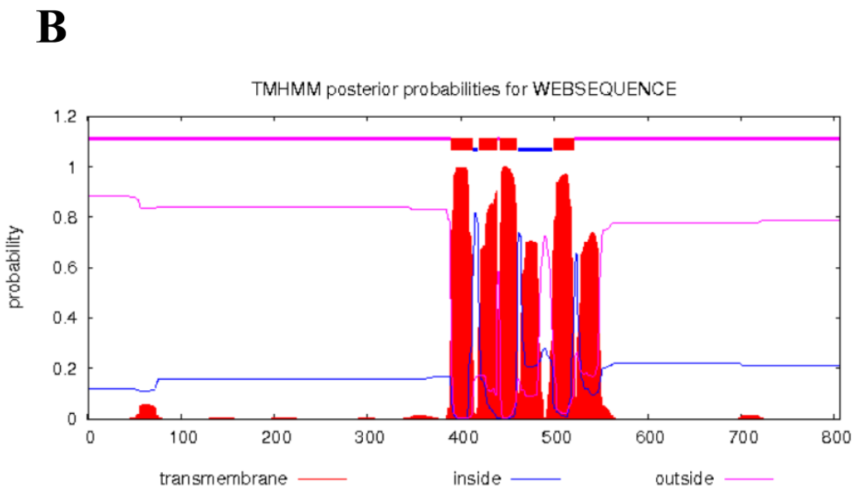

Fig. S1. TMHMM transmembrane prediction model for (A) $TMP_{TP901\text{-}1}$ wild type and (B) $TMP_{TP901\text{-}1}$ Δ1-10 highlighting that the second TMD contains only one membrane-spanning region (B) in comparison to the three observed in the native protein (A).
